# Using mouse transgenic and human stem cell technologies to model genetic mutations associated with schizophrenia and autism

**DOI:** 10.1098/rstb.2017.0037

**Published:** 2018-01-29

**Authors:** David St. Clair, Mandy Johnstone

**Affiliations:** 1 Institute of Medical Sciences, University of Aberdeen, Foresterhill, Aberdeen, UK; 2 Division of Psychiatry, University of Edinburgh, Royal Edinburgh Hospital, Edinburgh, UK; 3 Centre for Genomic and Experimental Medicine, Institute of Genetics and Molecular Medicine, University of Edinburgh, Edinburgh, UK; 4 Centre for Clinical Brain Sciences, University of Edinburgh, Edinburgh, UK

**Keywords:** Schizophrenia, autism, mouse and iPSC models

## Abstract

Solid progress has occurred over the last decade in our understanding of the molecular genetic basis of neurodevelopmental disorders, and of schizophrenia and autism in particular. Although the genetic architecture of both disorders is far more complex than previously imagined, many key loci have at last been identified. This has allowed *in vivo* and *in vitro* technologies to be refined to model specific high-penetrant genetic loci involved in both disorders. Using the *DISC1*/*NDE1* and *CYFIP1*/*EIF4E* loci as exemplars, we explore the opportunities and challenges of using animal models and human-induced pluripotent stem cell technologies to further understand/treat and potentially reverse the worst consequences of these debilitating disorders.

This article is part of a discussion meeting issue ‘Of mice and mental health: facilitating dialogue between basic and clinical neuroscientists’.

## 1. Introduction

Schizophrenia (SCZ) and autism (ASD) are two of the most important neurodevelopmental disorders encountered in routine clinical psychiatric practice. Both are diagnosed on the basis of clinical history, symptoms and behaviour. These include SCZ-positive symptoms, such as hallucinations, delusions and thought disorder, and SCZ-negative symptoms such as social withdrawal anhedonia and poverty of thought: there are also a range of cognitive abnormalities especially of attention, memory and executive function. ASD is characterized by abnormalities of social communication and interaction and repetitive patterns of interests and behaviour. Unfortunately, in spite of intensive efforts spanning several decades, there are still no objective tests (biomarkers) in routine clinical psychiatric practice to assist with diagnosis of any psychiatric disorders including SCZ and ASD [1]. Although ages of clinical presentation of SCZ and ASD are normally early adult life and early infancy, respectively, both have at least in part neurodevelopmental origins, namely antecedents affecting brain development, and, in turn, predisposition to one or both disorders can occur at any point in the life cycle probably from conception onwards. There are also pre-conceptual intergenerational effects, the most studied being parental and grandparental age. Antecedents may be environmental or genetic/epigenetic or the effects of gene–environment (G × E) interactions. Environmental risk factors are often discussed in the context of a ‘stress-vulnerability’ aetiological model where early biological and psychological insults, occurring in both the pre- and postnatal periods, result in changes of gene and protein expression, and/or changes in the intracellular and extracellular milieu of the developing brain. For recent relevant reviews of environmental risk factors, see [2,3]. However, perhaps, the most intriguing finding to emerge from epidemiological studies is that SCZ and ASD appear to share a remarkable number of environmental risk factors [4,5]. A similar pattern of overlapping genetic risk profiles for SCZ and ASD will be discussed below.

## 2. Familiarity

SCZ and ASD are both strongly familial neuropsychiatric disorders. The evidence in SCZ comes from multiple twin family and adoption studies and points to a heritability of up to 80% with monozygotic concordance of 40–50% [6,7]. There have been many fewer twin and family studies of ASD and surprisingly no adoption studies. Earlier twin studies suggested heritability as high as 80–90% for ASD with little contribution from the environment [8]. Newer studies of MZ twins have yielded concordance rates of less than 50%, with lower concordance for dizygotic twins, suggesting that both genes and environment play roles in the development of ASD [9]. The current consensus is that up to 40–50% of variance is determined by environmental factors [2].

Early linkage and candidate gene mapping studies of SCZ and ASD have yielded little in the way of findings that have stood the test of time. The most studied are *disrupted in schizophrenia one* (*DISC1* gene) identified by cloning the breakpoints of a balanced 1 : 11 chromosomal rearrangement associated with multiple cases of mental illness including SCZ in a large Scottish pedigree [10], chromosome 22 deletion syndrome associated with a range of severe neurodevelopmental disorders [11], fragile × syndrome [12], Rett syndrome [13] and rare cases of ASD with mutations of neuroligin genes [14].

Our understanding of the genetic architecture of neurodevelopmental disorders expanded enormously with the advent of methods for systematic interrogation of DNA across the whole genome, first through genome-wide association studies (GWAS) and more recently whole exome and whole genome sequencing. It allows us to detect association with rare (less than 1%) high-penetrant genetic lesions including copy number variants (CNVs) and association with common low-penetrant genetic risk factors identified using single-nucleotide polymorphism (SNP) microarrays. These latter common low-penetrant risk factors have odds ratios approximately 1.0–1.2. Rare low-penetrant genetic lesions also exist, but sample sizes required for their detection with reasonable statistical supporting evidence are far beyond those currently available worldwide; a similar problem confounds potential genome-wide studies of gene/gene interactions/epistasis [15]. It is also possible to examine non-statistically significant common variants for association as a whole so-called polygenic liability risk [16]. This latter approach does not, however, help with selection of individual gene targets for *in vivo* or *in vitro* modelling.

### (a) Rare variants

The genetic findings in both SCZ and ASD are broadly similar. In both disorders, there is enormous genetic heterogeneity, but only a small proportion (5–10%) of the overall genetic risk results from rare high-penetrant genetic mutations including CNVs. Many of these latter loci (causing deletions or duplications of stretches of DNA) show pleiotropy, i.e. they display a range of clinical phenotypic abnormalities that include ASD, intellectual impairment, SCZ and epilepsy [17]. This means that there is considerable overlap of high-penetrant loci between SCZ and ASD. Many mutations, especially in ASD, have arisen *de novo* and are not found in the parents of the affected proband [18–21]. This reflects the fact that they are heavily selected against due to the reduced fecundity associated with neurodevelopmental disorders. In ASD, this is so pronounced that it is almost impossible to find families with ASD in more than two generations. In SCZ, familial cases are more common but with high-penetrant loci, the effects of reduced fecundity are also clinically observable. This was elegantly demonstrated in the Icelandic population where it was possible to examine formally family inheritance patterns of recurrent non-*de novo* SCZ-associated CNVs. Although recurrent CNVs have high mutation rates due to non-allelic homologous recombination, they are eliminated fast by negative selection and seldom survive more than two or three generations [22]. Around 800 rare loci are reported in ASD (far fewer in SCZ), but the evidence to support their causal involvement varies enormously and in only a few dozen including recurrent CNVs is there statistical evidence of genetic association [19]. Among these genes are *NGLN4X* [14], *SHANK3* [23,24], *NRXN1* [25,26], *SHANK2* [27], *CNTN4* [28–30] and *CNTNAP2* [31,32]. The findings in SCZ are broadly similar [33–35].

### (b) Common variants

Initial genome-wide SCZ SNP association studies, involving several thousand cases and controls, yielded only two or three loci that meet statistical significance (*p* < 1 × 10^−7.5–8^), the precise significance level depending on the number of tests performed [16,22]. However with increased sample sizes to around 150,000 individuals, over 100 loci were reported to meet genome wide significance [36] with additional loci being subsequently reported [37]. Although multiple common low-risk variants are reported associated with ASD, to date no loci for ASD have consistently met criteria for genome-wide significant association; this is probably the result of inadequate sample sizes. There are a number of excellent articles discussing gene/gene interactions/epistasis [15], SCZ epigenetics [38] and modelling of polygenic risk [16,39]. In particular, two earlier studies highlight the potential of being able to elucidate a better understanding of the effects of regulatory polymorphism on the expression of genes essential to mental health [40,41]. Furthermore, the identification of these regulatory determinants will, in turn, permit critical insights into the role of epigenetic factors such as DNA methylation that are known to influence gene expression. To date, however, there are very few instances where specific low penetrance loci for ASD or SCZ have been deemed worthy of modelling in animals or human-induced pluripotent stem cell (hiPSC).

### (c) Missing heritability

The majority of genetic risk for both SCZ and ASD is still to be elucidated and is likely to involve many more rare high- and low-risk factors, common low-risk factors, epistasis and epigenetic interactions, the so-called missing heritability. Their tiny effect sizes represent a formidable challenge: what sort of clinical or behavioural phenotype if any should one expect to find? A recent ‘omnigenic model’ has proposed that gene regulatory networks are sufficiently interconnected, such that all genes expressed in disease-relevant cells are liable to affect the functions of core disease-related genes and that most heritability can be explained by effects on genes outside core pathways [42]. The genes we have chosen to discuss here are likely to affect the function of core pathways and so are likely to provide insights into wider populations of patients with these disorders, even although the majority of patients are not enriched for high-impact variants.

## 3. Modelling: from mice to men

Two of the most important methods for attempting to model neurodevelopmental disorders are genetically modified animals, especially rodent models, and *in vitro* modelling using hiPSCs differentiated into neuronal precursors and, in turn, to three-dimensional organoid systems. The advantages and disadvantages of the two methods are elegantly described elsewhere [43], but are summarized below with modifications. It cannot be stressed enough however that the full benefits of modelling studies are predicated on knowing what phenotype to expect and this depends on careful and deep phenotyping of patients and individuals with mutations at the specific loci under investigation. The enormous genetic heterogeneity encountered in SCZ and ASD as well as locus pleiotropy makes predictions of expected phenotype from population findings alone much less satisfactory.

### (a) Animal models: the pros and cons

Animal models of disruption exist for almost all human genes. Coding regions of the genome are especially well preserved and easier to model, whereas non-coding DNA, including regulatory elements, show poor conservation across species. The mouse genome is almost as well characterized as the human and murine models have become relatively cost effective, straightforward to produce, and amenable to study at molecular, cellular, circuit and behavioural levels. The advantages of rat models are usually outweighed by the costs of their generation and maintenance. The highest-throughput and least-expensive models include zebrafish (*Danio rerio*) and fruit fly (*Drosophila melanogaster*) [44], but obviously these are unsuitable for modelling more complex human behaviours. Care must be given also to which mouse strain is used as genetic background effects are potential confounders. However, there are a number of very obvious limitations and drawbacks when using such models to study neuropsychiatric disorders. Although there are established batteries to phenotype core features of ASD in mice [45], mice exhibit profound differences in social behaviour from humans and furthermore, even within mouse studies, variations in laboratory environments impose further variance. How these truly reflect the human condition is debatable. Interpreting SCZ like phenotypes in mice, including complex symptoms such as paranoia and delusional beliefs, is even more challenging: they can only be inferred indirectly from disordered mice behaviour, a major limitation of modelling schizophrenia in animals.

### (b) Human *in vitro* stem cell models: advantages and limitations

Human iPSC technologies are allowing researchers to interrogate human cortical development in health and disease and provide unlimited platforms of mature neuronal and glial cellular subtypes and co-cultures for downstream studies such as cellular physiology, phenotypic screening, and for drug development and screening. Such human iPSC models confer a number of advantages including the fact that it is possible to model for both coding and non-coding variants and, in fact, it is also possible to model for disease without actually knowing the causal genetic factor [46]. Clearly, though knowing the causal/contributory variants confers an advantage to translational studies and a greater understanding of putative mechanisms of disease [47]. It is possible to study the effects of genomic mutations on brain development and in neuropsychiatric disorders using clustered regularly interspaced short palindromic repeats (CRISPR) gene-editing technologies. Proteomics, transcriptomics, signalling and cell biology analysis of isogenic-mutant paired lines at the neuronal stem cell and differentiated neuron cell state offer unique opportunities. However, limitations include heterogeneity and reproducibility issues arising from multiple sources, including culture methodology and differences in lines and clones used. Furthermore, these hiPSC cultures produce immature fetal-like neurons, limiting their potential to properly model later developmental stages. This, however, has become less of an issue as it is now possible to mature cells by co-culture and also using advanced organoid cultures [48,49], discussed further below.

### (c) Towards three-dimensional cellular systems: growing brain organoids

An organoid is a multicellular collection of cells that self-organizes and develops from stem cell progenitors to resemble the structure and function of an organ *in vivo* [50]*. In vitro* models of the developing brain such as three-dimensional brain organoids offer an unprecedented opportunity to study aspects of human brain development and disease, in particular the ability to follow development over time. Neuronal migration, cortical lamination, projection patterns and circuit-level organization are difficult to model in two-dimensional cultures. Tissue engineering and three-dimensional organoid cultures will enable the study of some of these phenotypes. As mentioned earlier, rodent models have been heavily used to study the cellular function of many of the genes implicated in these disorders, especially those genes which are proposed to have an important role in fundamental neurodevelopmental processes such as cerebral cortex organization. However, cortex development and organization is very different in rodents compared to humans, so unsurprisingly neurodevelopmental diseases cannot be consistently recapitulated in animal models. This is all about to change as over the past few years there have been further cutting-edge advances in developmental neurobiology: we can now grow three-dimensional cerebral organoid cultures from patient-derived stem cells to study the early events of human brain development. Proof-of-principle studies using human pluripotent stem cell-derived three-dimensional organoid cultures have allowed researchers to model human brain development and microcephaly in a dish [51]. These ‘cerebral organoids’ develop various discrete brain regions including a cerebral cortex that produces functional cortical neuron subtypes capable of displaying spontaneous synaptic transmission and producing action potentials. Subsequent studies have also shown that it is possible to develop region-specific identities, including neocortex [52], telencephalon [53], cerebellum [54], neural tube [55], pituitary [56], hippocampus [57], optic-cup [58] and retina [59]. Through altering specific culture conditions, it is possible to differentiate iPSC and embryonic stem cells (ESC) into a range of neuronal [60] and glial subtypes, including GABAergic interneurons and glutamatergic neurons [61,62], dopaminergic neurons [63], motoneuron [64] and glial progenitors [65,66].

**Figure 1.**
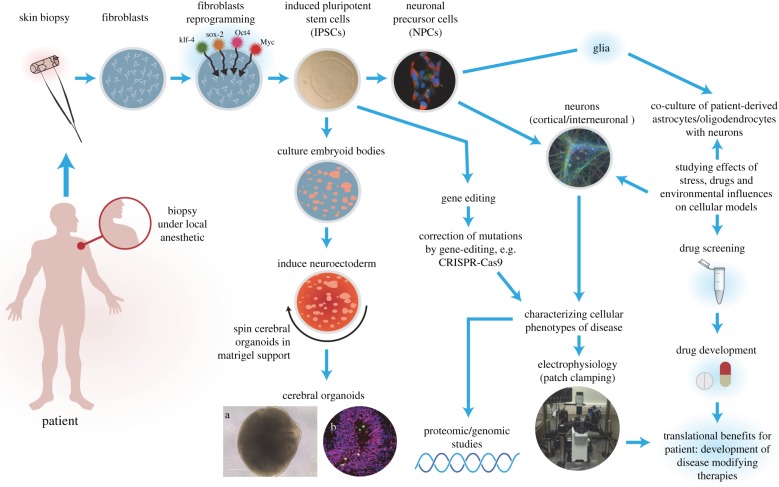
Overview of human iPSC model systems to study SCZ and ASD. Human iPSCs are generated by reprogramming fibroblasts from skin biopsies from volunteers using a variety of techniques, most commonly using standard Yamanaka factors, delivered in non-integrative episomal vectors. Other starting cellular materials can be used such as keratinocytes from hair or from peripheral blood mononuclear cells. Once generated and extensively tested, the hiPSCs can be used to either make neuronal precursor cells or glial precursor cells (e.g. oligodendrocyte precursors) or grown and lifted to make three-dimensional organoids as shown in frames (a) phase bright image of a cerebral organoid at two months of age and frame (b) shows an organoid that has been sectioned and stained with antibodies to Pax6 and phospho-histone H3, clearly demonstrating a ventricular zone. The cellular platforms generated can then be used for further downstream studies including electrophysiology, transcriptomic and proteomic studies, drug screening as well as morphological studies, and co-culturing with other cell types. In addition, the hiPSCs can be gene-edited using CRISR–Cas9 to attempt to rescue phenotypes observed.

Most protocols adopted to generate cerebral organoids depend on step-wise establishment of spatio-temporal strategies using human ESC or iPSC (figure 1). The first stage depends on the re-aggregation of iPSCs or ESCs in low-adhesion conditions such as those provided by serum-free embryoid body (EB) protocols, and allowing the cells enough time to proliferate and expand [67,68]. During this initial stage, the stem cells maintain pluripotency and the EBs that form exhibit all three germ layers (ectoderm, mesoderm and endoderm). The next stage involves neural induction where the goal is to drive differentiation to neuroectoderm formation. During these early stages, the initial organoids formed display apical–basal and dorsal–ventral polarity and further induction can promote regional identity such that it is possible to produce region-specific organoids [49]. The human cerebral cortex is a well-defined structure with six layers of neurons: superficial and deeper layers are connected to one another, yet have distinct structural and functional projections and fates [69,70]. One of the greatest challenges in the development of the human cerebral cortex is the assembly of circuits composed of glutamatergic neurons, generated in the dorsal forebrain (pallium), and GABAergic interneurons arising in the ventral forebrain (subpallium). However, it has recently been shown for the first time using a three-dimensional differentiation approach using hiPSC to specify neural spheroids and assemble these *in vitro* to model salutatory migration of human interneurons towards the cerebral cortex and functionally integrate into microcircuits [49].

Organoid cultures are, however, not without limitations: spontaneous self-organization of cerebral organoids in culture generates significant heterogeneity in cell type and structure, with prolonged neotany in development and differentiation limits their utility to early studies of brain development. There are also challenges with scalability. However, with modifications to culture systems such as the use of mini-reactors [71] and microfluidics [72] combined with improved seeding technologies (e.g. laminin-coated nanoparticles) [73], it is possible to scale-up, improve consistency and robustness, and reduce associated costs plus provide higher throughput for drug screening. In this regard, iPSC-derived two-dimensional and three-dimensional model systems hold potential in future to screen drug targets for pharmaceutical development (figure 1). Fundamentally, however, it is a human *in vitro* system, and as such *in vivo* connectivity and external milieu are not preserved, thus findings may not precisely translate to *in vivo* biology experienced during human fetal brain development. It is also important to remember that the human brain develops both *in utero* and during the postnatal period in an environment with inputs via sensory systems as well as from neighbouring brain areas which collectively helps to shape the cellular environment and circuits that develop. Obviously, *in vitro* culture systems cannot recapitulate this degree of complexity other than ambient fluctuations in temperature, pH and chemical gradients. Furthermore, the lack of neuromodulatory inputs to synaptic function may preclude our ability to precisely study the effects of systems such as the monoaminergic system in neuropsychiatric disorders and limit their utility in drug development. Until recently, another major drawback of organoids to model neurodevelopmental disorders was the unanswered questions as to what extent they truly modelled regional complexity, cellular diversity and circuit functionality of the brain. Gene expression analysis in over 80 000 individual cells isolated from 31 human brain organoids has shown that organoids generate a broad diversity of cells, which are related to endogenous classes, including cells from the cerebral cortex and retina [74]. Some caution should be held, however, as to the relative quantities of the different cell types generated in these organoid systems and to what extent this reflects the quantities in the human developing embryonic and fetal brain. In the Quadrato *et al*.'s study [74], only two of 10 cell clusters analysed were found to contain neurons from the cerebral cortex, accounting for approximately 20% of cells examined, somewhat less than what might be expected *in vivo*. Furthermore, these two cell clusters were found in only 32% and 52% of all organoids examined and within these populations approximately half of the cells expressed the radial glial marker PAX6 after six months, reflecting that they could not truly be classified as wholly mature neurons. However, this study has allayed fears that organoid cultures are limited by immaturity as a proportion of the cells do appear more mature than has been seen previously in culture. This team also elegantly demonstrated that neuronal activity, within the organoid, could be controlled using light stimulation of photosensitive cells which provides further opportunity for the coupled use of optogenetics to probe the functionality of human neuronal circuits and specifically model higher-order functions of the human brain, such as cellular interactions and neural circuit dysfunctions related to neurodevelopmental and neuropsychiatric pathologies.

## 4. Selection of loci for modelling of neurodevelopmental disorders

### (a) Single-nucleotide polymorphism-associated loci

In spite of their large numbers and widespread involvement in SCZ and ASD, there have been few attempts to model individual common low-penetrant SNP-associated loci using either animals or human iPSC technologies. This is unsurprising. The vast majority of SNPs significantly associated in GWAS are located outside gene-coding regions and, in many instances, often a considerable distance from the nearest coding region. Most effort has, therefore, concentrated on attempting to fine map putative functional variants presumed to be in linkage disequilibrium with the GWAS-associated SNPs. This is paralleled by *in silico* bioinformatic investigations using pathway analyses/gene ontology studies to try to obtain further corroboration of their functional significance. To date, success has been very limited [33]. Fortunately, successful studies designed to ascribe regulatory functionality to directly associated SNPs, or those in linkage disequilibrium, using comparative genomics and CRISPR-modified preclinical mouse models are well underway. These studies promise to develop a better understanding of the effects of regulatory polymorphism on the expression of genes essential to mental health. Furthermore, the identification of these regulatory determinants will, in turn, permit critical insights into the role of epigenetic factors such as DNA methylation that are known to influence gene expression.

Two modelling attempts are worthy of note.

(1) The very strong allelic association of SCZ to the major histocompatibility complex region of chromosome 6 prompted detailed exploration of the putative involvement of complex variation at the complement component 4 candidate gene. In mice, the authors showed that some patterns led to excess synaptic pruning [75], a dynamic process proposed to rid the brain during the development of wasteful neural connections and strengthen others, and proposed to be a reason why brains from patients with SCZ have fewer synaptic connections in multiple brain regions [76].
(2) The strong association of SNPs within CACNA1C with autism, bipolar disorder and schizophrenia [36,77] has been investigated functionally.

The risk-associated genotypes appear to affect RNA abundance but results are inconclusive. CACNA1C has several dozen exons, with multiple transcripts and promoters. The locus is also independently associated with *de novo* Mendelian dominant exonic mutations responsible for Timothy syndrome (TS), a neurodevelopmental disorder which has features of ASD. The most interesting findings have emerged from studying hiPSCs from individuals with TS [49,78]. Building on previous work that showed in rodents that L-type calcium channel (LTCC) genes play a critical role in interneuron migration [79]. Birey *et al*. [49] found that cortical interneurons derived from patients with TS display a cell-autonomous migration defect whereby they move more frequently but less efficiently [49]. What is more the TS interneuron defect is rescued by pharmacologically manipulating LTCCs.

### (b) Rare highly penetrant genetic mutations

The selection of which high-penetrant genetic mutations to model in mice or using hiPSC technologies poses separate challenges from common low-risk SNP-associated loci.

*Causative or non-causative?* Often the mutations are so rare that statistical evidence of association with the disorder is lacking. This is less of a problem in SCZ where linkage with the mutation in multiplex families is often available for additional corroboration. Also through PGC and other consortia, DNA from many thousands of cases is available for interrogation to try to identify additional mutations at the locus of interest. In ASD, where *de novo* mutation is more common, corroborating data from multiplex families is usually not available. It can be argued that *de novo* mutation itself may support a causative role in a disorder where the absence of familial cases is due to negative selection. A word of caution is merited. It must be borne in mind that each individual harbours approximately 60–100 *de novo* events [80], and deciding which/if any are causative is not a trivial problem, especially if it has implications for genetic counselling. Often, therefore, one of the main purposes of modelling is to try to demonstrate a causative mechanism that may result in the disorder under investigation. This especially applies where the gene is not an obvious candidate for the disorder under investigation, e.g. complement component 4 discussed above. In the case of rare variants, biology *does* have a role in both establishing a genetic association and later in understanding its role [81].

In some cases, the statistical and/or circumstantial evidence for involvement of the locus with the disorder is sufficiently compelling that modelling in mice and/or hiPSCs justifies the time and cost. Many such loci are currently being examined using animal modelling and hiPSC technologies. In these circumstances, the main questions concern what sort of phenotype to expect at the different levels of analysis and obviously also how to decide the precise nature of the modelling itself. The authors have been fortunate to have been involved with the identification and/or analysis of several loci that meet such criteria. These we discuss in more detail below. They are (i) *DISC1* and a key interactor *NDE1* and (ii) *CYFIP1* and *EIF4E* genes which, with *FXMR*, encode for a single molecular complex responsible for translation including at the synapses in the brain.

## 5. Disrupted in schizophrenia one

*DISC1* is a major vulnerability factor for a wide range of chronic mental illnesses, including SCZ [82]. *DISC1* was first isolated by cloning the breakpoints of a 1 : 11 balanced translocation co-segregating with major psychiatric disorders in a large Scottish pedigree [10,83]. Within this one family, the logarithm of the odds (LOD) score for SCZ alone met stringent genome-wide significance, while for SCZ plus bipolar disorder and major depressive disorder, it substantially exceeded genome-wide significance (multipoint logarithm of odds = 7.1). A second wave of follow-up confirmed these original findings [84]. Further evidence supporting the involvement of *DISC1* in mental disorders has been more recently debated [81,85].

DISC1 expression in the brain is particularly high in the hippocampus during neurogenesis and remains high in the adult dentate gyrus, olfactory bulb and limbic regions [86,87], and it appears that DISC1 regulates important developmental processes such as neuronal migration, integration [88], synapse formation and neuronal stem cell maturation [87,89–91]. DISC1 is thus critical for neurodevelopment and normal adult neuronal function. In addition, transgenic or mutant mice with impaired DISC1 function show brain morphological changes, deficits in neural circuits, working memory impairment and behavioural traits related to SCZ and also bipolar disorder [92]. One of the more interesting of the mice transgenic models, denoted Disc1tr, expresses two copies of truncated Disc1 encoding the first eight exons generated using a bacterial artificial chromosome (BAC) [93]. With this partial simulation of the human situation, they discovered a range of phenotypes including a series of novel features not previously reported. Disc1tr transgenic mice display enlarged lateral ventricles, reduced cerebral cortex, partial agenesis of the corpus callosum and thinning of layers II/III with reduced neural proliferation at mid-neurogenesis [93]. Parvalbumin (PV+) GABAergic neurons are reduced in the hippocampus and medial prefrontal cortex, and displaced in the dorsolateral frontal cortex. In culture, transgenic neurons grow fewer and shorter neurites. Behaviourally, these transgenic mice exhibit increased immobility and reduced vocalization in depression-related tests, and impairment in conditioning of latent inhibition. The BAC mouse model uses the full mouse genomic sequence and natural promoters. This may be responsible for the considerable SCZ reminiscent brain pathology observed in this study compared to other studies using more artificial constructs.

It is still not clear the mode of action of the t1 : 11 mutation. Haploinsufficiency seems most likely. No truncated DISC1 protein has ever been identified, suggesting elimination of mutated RNA by non-sense-mediated decay. It has also been shown that transient knockdown of DISC1 by *in utero* electroporation in mouse, in the pre- and perinatal stages, specifically in a lineage of pyramidal neurons mainly in the prefrontal cortex, leads to selective abnormalities in postnatal mesocortical dopaminergic maturation and behavioural abnormalities associated with disturbed cortical neurocircuitry after puberty [94]. Nevertheless, a dominant negative mode of action from mutated DISC1 protein dimerizing with the wild-type cannot be ruled out. What is clear is that the mutations reported in *Disc1* do seem to alter the structural organization of the DISC1 protein [95].

The molecular and genetic mechanisms that are involved in biological alterations can often be modelled in *Drosophila* or zebrafish [44]. DISC1 causes associative memory and developmental defects and disruption of sleep rhythms in *Drosophila* [96,97]. In zebrafish studies, *DISC1* variants were first identified from patient pools and tested in *Disc1* loss-of-function (LOF) mouse embryos to determine which could and which could not rescue neuronal progenitor proliferation. When they were injected in *disc1* LOF zebrafish embryos, the variants that showed maintenance or loss of activity in mice exhibited similar patterns in rescuing or not, respectively, brain ventricle and axon tract defects in zebrafish embryos [98]. These results emphasize the conservation of variant function between species, and indicate that a much higher number of variants can be analysed in zebrafish than is feasible in the mouse.

It has also been possible to generate isogenic hiPSCs with an engineered disease-relevant disruption of *DISC1*, which affects neural progenitor cells (NPCs) proliferation, baseline wingless-type mouse mammary tumor virus integration site signalling and expression of NPC fate markers such as FOXG1 and Tbr2 [99]. Ming & Song's group have since generated hiPSCs from four members of an American family in which a frameshift mutation of *DISC1* co-segregated with major psychiatric disorders [100] and furthermore produced different isogenic iPS cell lines via gene editing [101]. In an elegant series of experiments, they showed that mutant DISC1 causes synaptic vesicle release deficits in hiPSC-derived forebrain neurons [102–104]. Mutant DISC1 depleted wild-type DISC1 protein and, furthermore, dysregulated expression of many genes related to synapses and psychiatric disorders in human forebrain neurons, providing new insights into the molecular and synaptic etiopathology of psychiatric disorders [101]. Although similar studies have not yet been published from the Scottish DISC1 family, it will be interesting to see whether synaptic dysregulation is also evident in neurons derived from these hiPSCs.

Unlike in ASD, SCZ psychosis can be thought of as a neurodevelopmental disorder with psychosis as a late stage of illness, even though several population-based studies indicate that the problems are evident much earlier [105]. In this model of SCZ, Insel proposes that reduced myelination could alter connectivity in SCZ. There are multiple studies showing white matter changes in SCZ (reviewed in [106,107]) and specifically in the DISC1 family [108]. It will be possible using hiPSC from the DISC1 family to generate oligodendrocyte and astrocytes to study the impact of glia on the pathophysiology. Insel also argues that the trajectory of cognitive development in children developing SCZ could include reduced elaboration of inhibitory pathways and excessive pruning of excitatory pathways leading to an altered excitatory–inhibitory balance in the prefrontal cortex. In this regard, it will also be interesting to now use hiPSC-derived GABAergic interneurons from the DISC1 families to specifically look for deficits in inhibitory interneuron activity and *N*-methyl-d-aspartate receptor expression. Protocols are now available to generate GABAergic inhibitory interneurons from hiPSC [109], which can be matured in culture to generated PV+ interneurons for the electrophysiological study of these cell types *in vitro*. Furthermore, as discussed earlier, Birey *et al*. [49] have recently generated three-dimensional spheroids from hiPSC that resemble either the dorsal or ventral forebrain and contain cortical glutamatergic or GABAergic neurons [49]. This is a seminal study as it demonstrates for the first time that it is now possible to generate organoids/spheroids with network activity: these subdomain-specific forebrain spheroids can be assembled *in vitro* to recapitulate the salutatory migration of interneurons observed in the fetal forebrain. These protocols will open the gates for the generation and studies of human forebrain spheroids from hiPSC from patients with other disease-associated mutations of SCZ and ASD.

## 6. Nuclear distribution E homologue 1

*NDE1* (*nudE Nuclear Distribution E homologue 1*) is a gene in which different mutations result in a wide range of human brain diseases including microcephaly [110], intellectual disability [111], ASD [112], attention-deficit hyperactivity disorder (ADHD) [113] and SCZ [114–116]. *NDE1* encodes a cytoskeletal protein localizing to the centrosome that participates in essential neurodevelopmental processes, including neuronal precursor proliferation and differentiation, neuronal migration and neurite outgrowth [117]. NDE1 is part of the lissencephaly-1/cytoplasmic dynein complex and as such participates in regulation of cell proliferation, migration and intercellular transport [118–132]. Cytoplasmic dynein is the main molecular motor moving towards the minus ends of microtubules, and is therefore responsible for carrying vesicles and other entities from axon tips towards the cell bodies of neurons (retrograde transport) [133–135].

Through protein–protein interaction, DISC1 regulates NDE1 function: evidence supports a shared binding domain for NDE1 and NDEL1 to DISC1, with opposite effects of the *DISC1* Ser704Cys mutation on binding patterns [129]. NDE1 and NDEL1 localize to the centrosome, and mutations in both genes result in defective neurogenesis and neuronal migration. This is proposed to arise from decoupling of the centrosome from the nucleus as a result of defective microtubule bundles connecting both organelles, and also from the proposed role, all three genes have in regulating the cell cycle and mitosis [122,136–138]. Furthermore, it has been shown that familial mutations in *NDE1* caused both severe failure of neurogenesis and a deficiency of cortical lamination (microlissencephaly) [110,139]. Elegant mouse studies have shown that while cortical lamination is mostly preserved, the mutant cortex has fewer neurons and very thin superficial cortical layers (II– IV) [138]. BrdU birthdating revealed retarded and modestly disorganized neuronal migration; however, more dramatic defects on mitotic progression, mitotic orientation and mitotic chromosome localization in cortical progenitors were observed in *Nde1* mutant embryos. Another *Nde1* mutant mouse study has demonstrated catastrophic DNA double-strand breaks concurrent with DNA replication, leading to p53-dependent apoptosis and reduced neurons in cortical layer II/III, and that this stalling of DNA replication in the *Nde1* mutants specifically occurred in mid-late S-phase [140]. More recently, knockdown in rat using *in utero* electroporation confirmed these findings and shows that *Nde1* effects are pronounced on premitotic nuclear migration with specific effects on radial glial progenitor cells and on primary cilia [141]. These studies elegantly demonstrate some of the mechanisms whereby haploid reduction of *Nde1* expression may cause more subtle neurodevelopmental phenotypes.

It should be highlighted that although *NDE1* does not appear as a top GWAS ‘hit’, deletions and duplications spanning NDE1 (on Chromosome 16p13.11) are among the most common CNVs in SCZ. CNVs in *NDE1* have also been found by others to associate with a range of phenotypically different neurodevelopmental disorders including intellectual disability [111], ASD [112], ADHD [113] and SCZ [111,114], which suggest that the locus contains dosage-sensitive gene(s) that may play a critical role in neurodevelopment. The deCODE genetics study of 4345 SCZ patients and 35 079 controls from eight European populations found a threefold excess of duplications and deletions at the 16p13.1 locus in SCZ cases, compared with controls with duplications being far more commonly found [115]. In a Scottish population sample, we found a fourfold excess of duplications at the 16p13.1 locus in SCZ patients compared with controls [116]. Significant sex differences in prevalence, course and severity have been described for a number of these conditions, but the biological and environmental factors underlying such sex-specific features remain unclear [142]. Rare SNPs in *NDE1* have also been shown to associate with SCZ susceptibility [143]. *NDE1* has also been identified as associating with psychosis proneness in a large Finnish birth cohort upon re-analysis of GWAS linkage data conditioned on a *DISC1-*associating risk haplotype [144]. Thus, consistent with current neurodevelopmental concepts in SCZ, the genetic and biological evidence for DISC1 and NDE1 provides evidence for a shared ‘risk’ pathway.

The underlying molecular mechanisms of the 16p13.11 microduplication, which despite being conserved across mice and human species, have remained elusive. Ingason *et al*. [115] subdivided the 16p13.1 region between 14.66 and 18.70 Mb (Human Genome Build 36) into three single-copy sequence intervals, denoted intervals I, II and III, each of which is flanked by sequences rich in low-copy repeats (LCRs). All duplications and deletions so far reported are contained within this region, with the most common breakpoints in the LCR clusters distal to interval I and proximal to interval II (so-called Dup I + II carriers) [115]. Dup I + II carriers showed the highest common odds ratio of all 16p13.11 microduplication carriers. The functional implication of these variants in mental illness and the mechanism of disease causation remain unknown, although the potential of investigating this in neuronal cell types derived from hiPSCs from specific patients hold much promise as has been shown in a proof-of-principle studies modelling SCZ and ASD using hiPSCs [43,46,47].

Despite the importance of studying neurodevelopmental disorders and because data from human embryonic tissue are scarce, there is a real challenge of finding an adequate model system. Rodent models have been heavily used to study the cellular function of *Nde1*, which revealed an important role of NDE1 protein in regulation of proliferation of neuronal progenitors and neuronal migration retardation. However, cortex development and organization is very different in animal models and humans. In particular, the outer subventricular zone, which is only present to a limited degree in rodents, is populated by a unique stem cell subset termed outer radial glia [145,146] that allow for the striking expansion in neuronal output and brain size seen in humans. Therefore, it is not surprising that neurodevelopment diseases cannot be consistently recapitulated in animal models. However, as discussed earlier, the beauty and utility of hiPSC-derived cerebral organoid will present a wealth of new possibilities to thoroughly study the role of *NDE1* in cellular proliferation, migration and differentiation, in real time, in the human cerebral cortex and allow the interrogation of genetic risk factors hypothesized to play important roles in human corticogenesis.

## 7. Cytoplasmic fragile X mental retardation 1–interacting protein

Chromosome 15q11.2 CNVs have emerged as prominent risk factors for various neuropsychiatric disorders, including SCZ, autistic spectrum disorder and intellectual disability [147]. 15q11.2 microdeletion (15q11.2 del) was identified as one of the most frequent CNVs associated with increased risk for SCZ [22], a finding subsequently confirmed in additional cohorts [114,148,149]. 15q CNVs are not as penetrant as other recurrent CNVs associated with neurodevelopmental disorders. They are, however, under negative selection [22] and even in normal subjects, and 15q11.2 del is associated with cognitive variation and changes in structural measures on MRI scanning [150]. 15q11.2 CNVs encompass four genes: non-imprinted in Prader–Willi and Angelman 1 and 2 (*NIPA1* and *NIPA2*), cytoplasmic fragile X mental retardation 1–interacting protein (CYFIP1) and *TUBGCP5.* While little is known about functions of these genes in mammalian neural development, *CYFIP1* has been shown to interact with *Rac1* [151], *FMRP* [152] and *eIF4E* [153]. Biochemical studies have also identified CYFIP1 as a regulator of the WAVE complex, consisting of WAVE1, WAVE2, Nap1 and Abi1, a complex known to regulate Arp2/3- mediated actin polymerization and membrane protrusion formation in non-neuronal cell lines [151,154,155]. The function of WAVE signalling in mammalian neurogenesis is not well understood. However, an elegant study has been published using stem cells from patients with 15q11.2 CNVs [47]. Yoon *et al*. [47] took a multifaceted approach to investigate why 15q11.2 CNVs are prominent risk factors for SCZ and ASD. Even in normal control subjects, carriers of the 15q11.2 deletion have cognitive deficits and structural changes on MRI scanning raising questions about how this genetic variant brings about these changes in the carriers. They showed that hiPSC-derived neural progenitor cells carrying 15q11.2 microdeletions exhibited deficits in adherens junctions and apical polarity resulting from haploinsufficiency of *CYFIP1* [47]. Furthermore, they showed that deficiency in CYFIP1 and WAVE in the developing mouse cortex affects radial glial cell migration causing ectopic localization outside of the ventricular zone [47]. Targeted human genetic association analyses revealed an epistatic interaction between CYFIP1 and WAVE signalling mediator actin-related protein 2 and risk for SCZ. Therefore, by integrating human neural stem cells, *in vivo* animal modelling and targeted human genetic association studies, a mechanistic understanding of how 15q11.2 microdeletions affect neural development has been uncovered.

## 8. Eukaryotic translation initiation factor 4E

*Eukaryotic translation initiation factor 4E (EIF4E)* is the rate-limiting component of eukaryotic translation initiation and plays a key role in learning and memory through its control of translation within the synapse. EIF4E-mediated translation is the final common process modulated by the mammalian target of rapamycin (mTOR), phosphatase and tensin homologue (PTEN) and fragile X mental retardation protein (FMRP) pathways, all of which are implicated in ASD [156,157]. Germline mutations in *PTEN* human homologue are present in 1–5% of patients with ASD, and *PTEN* knockout (KO) mice exhibit cognitive impairment and deficits in social interaction which are rescued by rapamycin [158]. Similarly, mutations in two tuberous sclerosis genes (*TSC1* and *TSC2*) cause ASD in a subset of patients with tuberous sclerosis. Mice with deletions of one copy of *TSC1* or *TSC2* genes also display deficits in synaptic plasticity and memory that are rescued by rapamycin. The mTOR/Eif4E pathway is hyperactivated in fragile × syndrome (F×S) patients, one of the leading genetic causes for ASD spectrum disorder. In F×S, a full mutation (greater than 200 repeats) leads to hypermethylation of *FMR1*, an epigenetic mechanism that silences *FMR1* gene expression and reduces levels of the *FMR1* gene product, FMRP. The absence of FMRP upregulates synaptic translation through failure of recruitment of CYFIP1, the EIF4E-binding protein [159]. The most well-characterized rodent model is the *Fmr1* KO mouse, which lacks FMRP protein due to a disruption in its *Fmr1* gene. These mice display a range of molecular, cellular, tissue and behavioural abnormalities consistent with the human phenotype, but the pattern and severity is variable depending among other things upon the strain of mouse [160].

Linkage of ASD to the EIF4E region on chromosome 4q was reported in genome-wide linkage studies [161] and was subsequently directly implicated in ASD [162]. In a boy with classic ASD, the authors observed a *de novo* balanced chromosome translocation between 4q and 5q and mapped the breakpoint site to within a proposed alternative transcript of EIF4E [162]. They then screened 120 ASD families for mutations in *EIF4E* and found two unrelated families where in each case both autistic siblings and one of the parents harboured the same single-nucleotide insertion at position 225 in the basal element of the *EIF4E* promoter. Electrophoretic mobility shift assays and reporter gene studies show that this mutation enhances binding of a nuclear factor and *EIF4E* promoter activity. These genetic observations implicate *EIF4E*, and more specifically control of EIF4E activity, directly in ASD. They raised the exciting possibility that pharmacological manipulation of *EIF4E* may provide therapeutic benefit for those with ASD caused by disturbance of the converging pathways controlling EIF4E activity.

These studies have been paralleled by molecular/cellular and animal studies aimed at elucidating the key downstream regulatory mechanisms responsible for so many upstream forms of ASD as well as mutations in *EIF4E* itself. In the brain, EIF4E activity is fundamental to the regulation of lasting alterations in synaptic strength or plasticity, and of long-term potentiation: these are important in learning and memory. Increased activity in these systems can lead to repetitive, perseverative behaviour patterns. In mice KO of EIF4E-binding protein (4E-BP2), an inhibitor of EIF4E leads to increased translation of neuroligins, also genetically implicated in ASD [14], as well as pathophysiological and behavioural abnormalities similar to those found in ASD. The phenotype was rescued by pharmacological inhibition [163]. In a separate study, direct overexpression of eIF4e in mice results in exaggerated cap-dependent translation and a range of repetitive and perseverative behaviours and social interaction deficits reminiscent of autism. They are accompanied by synaptic pathophysiology in medial prefrontal cortex, striatum and hippocampus. The autistic behaviours are corrected by intracerebral infusion of cap-dependent translation inhibitor 4EG1-1 [164]. In both studies, pharmacological normalization of EIF4E activity rectified many of the abnormalities observed in the mice [163,164]. These findings indicate that behavioural defects caused by exaggerated cap-dependent translation are not irrevocable and may be corrected well into adulthood.

## 9. Conclusion

The remarkable complexity of the genetic architecture of SCZ and ASD poses formidable challenges for clinicians and scientists aiming to find methods to diagnose, sub-classify, prevent and treat what were until recently considered incurable neurodevelopmental disorders. Over the last 10 years, however, a quiet revolution has been in progress: our understanding of key molecular pathways associated with SCZ and ASD has increased in leaps and bounds as have methods for modelling neurodevelopmental disorders in animals; this has been paralleled by the new opportunities presented by hiPSC technologies, especially when combined with CRISPR editing, three-dimensional organoid development and engraftment of *in vitro* technologies on to *in vivo* models; several instances now exist where the worst symptoms of human neurodevelopmental phenotypes can be arrested and/or reversed at least in non-human animal models and with *in vitro* hiPSC studies. This must surely be one of the most promising areas of current psychiatric research.

## Data accessibility

This article has no additional data.
